# A Digital Image-Based Phenotyping Platform for Analyzing Root Shape Attributes in Carrot

**DOI:** 10.3389/fpls.2021.690031

**Published:** 2021-06-16

**Authors:** Scott H. Brainard, Julian A. Bustamante, Julie C. Dawson, Edgar P. Spalding, Irwin L. Goldman

**Affiliations:** ^1^Department of Horticulture, University of Wisconsin-Madison, Madison, WI, United States; ^2^Department of Botany, University of Wisconsin-Madison, Madison, WI, United States

**Keywords:** *Daucus carota*, market class, high-throughput phenotyping, image analysis, diallel analysis, trait heritability

## Abstract

Root shape in carrot (*Daucus carota* subsp. *sativus*), which ranges from long and tapered to short and blunt, has been used for at least several centuries to classify carrot cultivars. The subjectivity involved in determining market class hinders the establishment of metric-based standards and is ill-suited to dissecting the genetic basis of such quantitative phenotypes. Advances in digital image acquisition and analysis has enabled new methods for quantifying sizes of plant structures and shapes, but in order to dissect the genetic control of the shape features that define market class in carrot, a tool is required that quantifies the specific shape features used by humans in distinguishing between classes. This study reports the construction and demonstration of the first such platform, which facilitates rapid phenotyping of traits that are measurable by hand, such as length and width, as well as principal component analysis (PCA) of the root contour and its curvature. This latter approach is of particular interest, as it enabled the detection of a novel and significant quantitative trait, defined here as root fill, which accounts for 85% of the variation in root shape. Curvature analysis was demonstrated to be an effective method for precise measurement of the broadness of the carrot shoulder, and degree of tip fill; the first principal component of the respective curvature profiles captured 87% and 84% of the total variance. This platform’s performance was validated in two experimental panels. First, a diverse, global collection of germplasm was used to assess its capacity to identify market classes through clustering analysis. Second, a diallel mating design between inbred breeding lines of differing market classes was used to estimate the heritability of the key phenotypes that define market class, which revealed significant variation in the narrow-sense heritability of size and shape traits, ranging from 0.14 for total root size, to 0.84 for aspect ratio. These results demonstrate the value of high-throughput digital phenotyping in characterizing the genetic control of complex quantitative phenotypes.

## Introduction

Carrot (*Daucus carota* subsp. *sativus*) is an economically and nutritionally important vegetable crop. Over 40 million metric tons of carrots are grown annually across the globe (FAO, 2020), and it is a significant source of pro-vitamin A in the human diet (Simon, 2000). Carrot root shape, which ranges from long and tapered to short and blunt, has been used for at least several centuries to classify carrot cultivars (Banga, 1957; Simon et al., 2008). Culinary practices and horticultural traditions have led to the establishment of the modern market classes based primarily on variation in root dimensions and shape differences, giving these component traits increasing economic importance. For example, cultivars that produce large and bulky roots grown for a full season—such as Danvers, Chantenay, and Berlicum—are typically used in canning, juicing, and other processing operations, while slimmer types such as Imperator, Kuroda, and Nantes are sold to fresh markets.

The shape differences that determine a root’s market classification range from obvious to very subtle. The distinctions between market classes frequently depend on subjective assessment of the curvature of the shoulder at the crown of the root, the variable degrees of tip fill, and specific combinations of these shape parameters and root dimensions. Furthermore, while the names of market classes would imply an assignment of cultivars to well-defined, discrete categories, all of the traits which define market class are quantitative, and intermediate cultivars may possess characteristics of more than one class. As a result, the subjectivity involved in assessing market class at present hinders the establishment of metric-based standards and are ill-suited to dissecting the genetic basis of such quantitative phenotypes. While advances in genetic resources in carrot have improved researchers ability to investigate the genetic architecture of key traits (Iorizzo et al., 2016; Que et al., 2019), this phenotyping bottleneck remains a key limitation in the study of root morphology.

Recent advances in measuring plant phenotypes through contour analysis of digital images have led to the development of methods for quantifying sizes and shapes of plant structures (Howarth et al., 1992; Horgan et al., 2001; Hameed et al., 2018), including those that are not captured by simple angles, lengths, widths, and their ratios (Iwata et al., 1998; Miller et al., 2017; Turner et al., 2018). Here we report the construction and demonstration of a platform which is capable of quantifying the shape features particular to carrot market class, and validate its performance in two experimental populations. First, a global collection of carrot accessions obtained from the USDA National Plant Germplasm System (NPGS) was used to test the performance of the phenotyping algorithms in a highly diverse context. In addition, a diallel mating design was constructed from pairwise matings between a defined set of inbred parental lines in order to estimate genetic variance components of key market class traits. Significantly, these analyses have provided the first quantitative description of a novel root trait in carrot—defined here as root fill—which accounts the majority of the shape variation across carrot genotypes. In addition, this study represents the first genetic characterization of shoulder and tip curvature, key aspects of market class in carrot. As a whole, this platform demonstrates the utility of digital contour analysis in the study of root morphology, and represents a robust, high-throughput workflow for future research.

## Materials and Methods

The digital image-based phenotyping methodology developed in this study followed a three-stage workflow: image acquisition, image pre-processing, and image analysis.

### Image Acquisition

Images were acquired using a DSLR camera with a 24 mm fixed-length lens, mounted above a template containing two 22.5 cm × 75 cm black-bordered rectangles. This facilitated imaging two roots simultaneously. Six fluorescent Interfit (Atlanta, GA) F5 lights provided overhead illumination in order to maximize contrast between roots and background, and eliminate shadow. Each rectangle was divided into an upper and lower portion by blue, 1.25 cm Gaffer’s tape. The upper portion contained a 100 mm scale bar, and a QR matrix barcode which encoded identifying information pertaining to the specific carrot root being photographed. The lower portion contained the corresponding root, placed on either a black felt or white vinyl background depending on the exterior pigmentation of the carrot. The top of the root was aligned to be parallel with the blue tape in order to precisely divide root and shoot growth.

The DSLR camera was connected via a USB cable to a computer running SmartShooter (Tether Tools, Phoenix, AZ) a tethered shooting application which allowed for high-resolution live previewing of the camera’s view-frame. This facilitated accurate positioning of carrots relative to the Gaffer tape. Upon image acquisition, SmartShooter wrote a raw and a lossless JPG image to disc at a user-specified “source” location.

**FIGURE 1:**
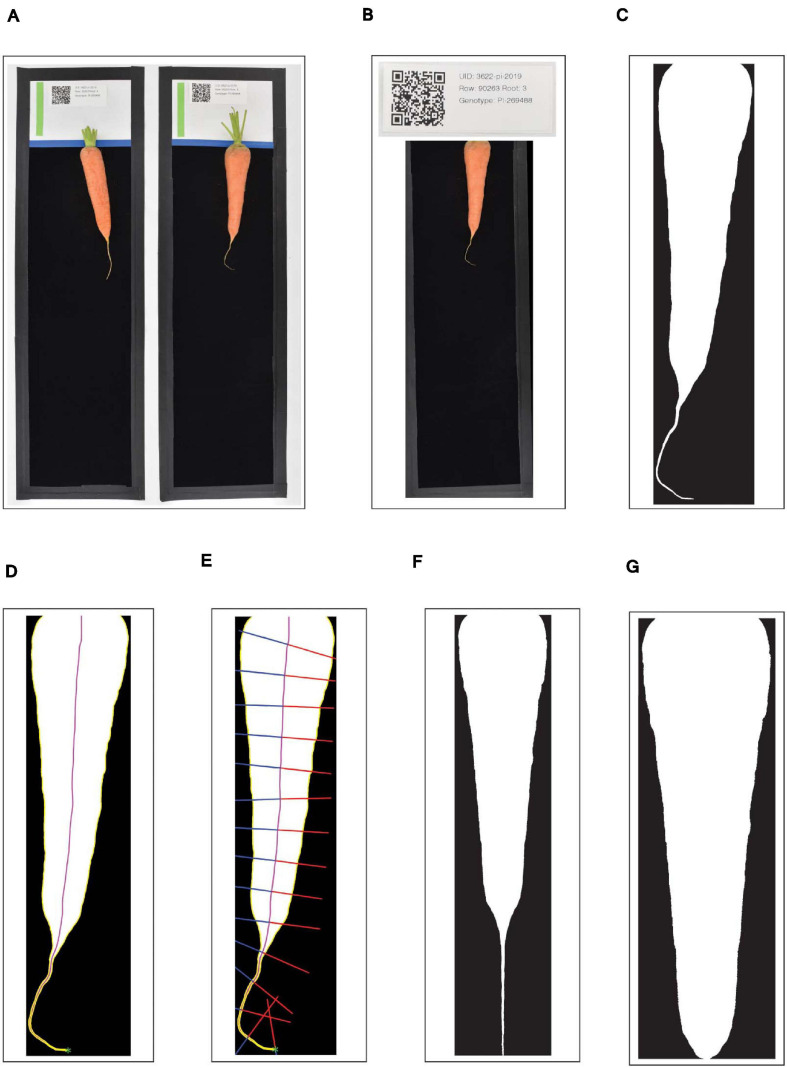
Workflow outlining the pre-processing of digital images of carrot roots. **(A)** Each black-bordered box within the overall image are identified; **(B)** QR codes within the upper portion of each box are scanned and the encoded text is displayed as a form of quality control; **(C)** carrot pixels are distinguished from background pixels to generate binary masks; **(D)** the midline of the carrot root is estimated by tracing a path from the carrot tip to the center of the shoulder, following the maximum of the smoothed Euclidean distance transform; **(E)** width measurements are made by sampling the binary mask normal to vectors tangent to the midline; **(F,G)** A random forest classifier is used to detect the point at which to “de-tip” any residual, unexpanded tap root.

A custom Python application then handled initial image processing and file management. This application runs inside of a Python v3.7.7 virtual environment in order to easily utilize a suite of open-source image-processing libraries. First, the watchdog library was used to detect each new JPG as it was created, and the lensfunpy wrapper for the C++ library lensfun was used to remove distortion due to the curvature of the lens (Figure 1A). Next, each black-bordered box within the image was identified using the Python bindings for the OpenCV library, and the QR code within the upper portion of each box was scanned using the bindings for the zbar library (Figure 1B). As a preliminary form of quality control, the click package was used to display the attribute-value pairs encoded by the QR code; when the user accepted these as accurate, the corresponding image was subsequently displayed within a browser window using Node.js, with a transparent overlay of the region detected as corresponding to the carrot root. This overlay was also generated using the OpenCV library: in brief, RGB images were converted to grayscale, a bilateral filter was applied to smooth the image while preserving edges, and a binary threshold was applied to generate the so-called “binary mask”—i.e., a black and white image in which white pixels designate the presence of carrot, and black pixels designate background (Figure 1C). If binary masks were visually judged as correctly identifying the root, they were then saved as high-resolution PNG files to a pre-specified “destination” directory path defined according to information encoded in each QR code (e.g., “Location/Year/Genotype”). All identifying information specific to each root was included in the PNG filename, in addition to a spatial resolution scaling parameter corresponding to the detected pixel length of the 100 mm scale bar.

### Image Pre-processing

Following image acquisition, pre-processing steps were performed to the standardized images, specifically by removing curvature and residual, unexpanded root tips. The straightening procedure was performed in MATLAB R2021a (MATLAB, 2021), and consisted of two stages. First, the midline of the carrot root was estimated, and second, widths were determined by sampling the binary mask along vectors normal to the tangent of this midline. To estimate the midline, the minimum distance between all points within the carrot root and contour of the carrot root was determined using the Euclidean distance-transform described by Maurer et al. (2003). Next, the tip of the carrot was found via an iterative algorithm that identifies points of maximum curvature within increasingly narrow segments of the root contour. Curvature at any particular boundary point is inversely proportional to the radius of a circle inscribed at this point. Thus, curvature values can be estimated by fitting a spline to a segment of the contour surrounding this boundary point. By parametrizing this spline, curvature (*K*) can then be calculated as:

$$K={\left\{\left(\frac{d\,x}{d\,t}\,\frac{d^{2}\,y}{d\,t^{2}}-\frac{d\,y}{d\,t}\,\frac{d^{2}\,x}{d\,t^{2}}\right)\,\left({\left[\frac{d\,x}{d\,t}\right]}^{2}+{\left[\frac{d\,y}{d\,t}\right]}^{2}\right)\right\}}^{-\frac{3}{2}}$$

where $\frac{d\,x}{d\,t}$ and $\frac{d^{2}\,x}{d\,t^{2}}$ represent the first and second derivative of *x* with respect to the parameter *t*, and $\frac{d\,y}{d\,t}$ and $\frac{d^{2}\,y}{d\,t^{2}}$ represent the first and second derivative of *y* with respect to the parameter *t*. Finally, following the procedure described by Miller et al. (2007), the midline was traced: starting at the tip, an ordered series of midline coordinates was sequentially determined by identifying the local maximum in the smoothed distance-transform, stepping in the direction of this local maximum, and then repeating the procedure. This “walk” along the maximum of the smoothed distance transform surface stably traces the midline, under the condition that all midlines must end by passing through the center of the shoulder of the carrot (Figure 1D).

In the second stage, the widths of the carrot along its length were calculated. Regardless of any degree of local skew or curvature in the root, the true width of the carrot at any point follows the vector normal to the tangent of the midline at that point. Thus, the binary mask was sampled along this vector, starting at the midline and moving outward in both directions until an intersection with the contour was reached (Figure 1E). This method of sampling prevented the inclusion of multiple segments of the carrot root in a single slice through its width, in cases where the tip of the carrot may curl back upon itself. A straightened version of the carrot was then obtained by arranging these widths into a single array (or “width vector”), all centered on their midpoints.

After straightening, a secondary pre-processing step was taken to remove any trailing, unexpanded portion of the taproot which extends past the tip of the carrot (Figures 1F,G). Any geometric definition of this cut point (e.g., on the basis of the derivative of width along the length of the root) is hindered due to extensive variation in tip fill.

A random forest classifier was therefore trained (using the sklearn Python library) with a subset of the images that were photographed both with their residual taproot attached, and with the taproot removed by hand. This model was subsequently used to detect the appropriate point at which to “de-tip” the straightened versions of the binary masks.

### Image Analysis

Following these pre-processing steps, images were analyzed for several different types of phenotypes. First, phenotypes that could be measured by hand, such as length, maximum width, and the width at different quantiles along the length of the carrot, were calculated for each image. These were determined by simply measuring the length of different slices through the straightened, de-tipped binary mask, and converting pixels to mm using each image’s scaling parameter.

Second, traits pertaining to individual roots that cannot be reliably measured by hand, such as total root size, tip angle, convex hull area of the shoulder, and curvature values of the shoulder and tip, were also calculated. Root size was defined as the total area of the binary mask. Tip angle was defined here as the interior angle formed by the line segments connecting the tip of the carrot to contour points located 10% up the length of the carrot toward its top, while shoulder hull area was the area encompassed by background pixels in the rectangular region bounding the top 10% of the carrot. Curvature values were computed at each point along the root profile in both the shoulder and tip regions (the first and last 50 contour points of the root, respectively) as described by Driscoll et al. (2012), using the equation described above. These vectors of curvature values in the shoulder and tip region were then summed, to generate a metric of total curvature, and decomposed using principal component analysis (PCA), to identify sources of variation in curvature values across a wide sampling of carrots.

Finally, in order to identify and quantify size-independent variation in the shape of the entire carrot root, PCA was also performed using the raw contour data of the entire width profile. In this case, the relevant covariance matrix was constructed using straightened, de-tipped masks that were first normalized such that all roots possessed equal lengths and maximum widths. Each root was represented by 1,000 standardized widths sampled evenly along the carrot’s length, and each width along a carrot’s length was divided by its maximum width, such that each carrot had a maximum width of 1.

### Validation of Accuracy and Reliability

To validate the accuracy of the pre-processing pipeline and phenotyping algorithms, 100 roots (10 each drawn from 10 carrot genotypes representing diverse market classes) were measured by hand prior to being photographed. Length measurements were made from the top of the shoulder to the point at which the unexpanded, residual tip of the carrot began using a tape measure, while maximum width was measured using calipers. Secondly, to determine the extent of the variance in phenotypes extracted from different 2D projections of a given root, 100 roots were photographed three times, with each root being rotated 45° following the acquisition of the first and second photograph.

### Visualization of the Phenotypic Correlates of Principal Component Analysis

In order to evaluate the performance of the principal component analyses, a diverse collection of carrot germplasm was grown at the Desert Research and Extension Center (Holtville, CA, United States) in 2019. This collection was obtained from the USDA NPGS, and consisted of a total of 683 cultivated accessions, comprising breeding lines, open-pollinated cultivars, and land races. These accessions represent a substantial amount of the global diversity in cultivated carrot, and thus provided an excellent basis for visualizing the variation captured by the first principal component of the normalized width profile, as well as the curvature in the tip and shoulder regions. Two replications of this collection were grown in 1 m plots, with 5–15 roots sampled randomly from each plot at harvest; in total, 8,687 roots were imaged in this analysis. The PC1 scores for each genotype were then estimated using a linear mixed effects modeled in which genotype and replicate were modeled as fixed effects, while subsampling within replicates was modeled as a random interaction term to account for unequal subsampling across genotypes; the resulting model is similar to an RCBD design with subsampling:

$$Y_{i\,j\,l}=\mu +G_{i}+R_{j}+G\,R_{i\,j}+\varepsilon_{i\,j\,l}$$

where *G_i* represents the *i*^*th*^ genotype effect, *R_j* the *j*^*th*^ environment effect, *GR*_*ij*_ the *ij*^*th*^ genotype × replicate interaction [with $G\,R_{i\,j}∼N\,\left(0,\sigma_{G\,R}^{2}\right)$] and ε_*ijl*_ the residual variance [i.e., variance among subsamples, with $\varepsilon_{i\,j\,l}∼N\,\left(0,\sigma_{\varepsilon }^{2}\right)$]. This model was fit using the lme4 package in R (R Core Team, 2020).

### PCA-Based Clustering Analysis of Market Classes

To test the discriminatory power of the phenotypes quantified by this pipeline, a clustering analysis was performed using length, maximum width, root fill, curvatures of the tip and shoulder, and aspect ratio (i.e., length divided by maximum width). Thirty five roots were drawn from each of five economically important and phenotypically diverse market classes: Chantenay, a short, bulky processing type; Imperator, a long and slender type used in baby carrot production; Danvers, a medium length, pointed type typical for fresh-market sale; Nantes, a medium length blunt type often used as a storage root; and Parisienne, a very short, rounded type often sold to specialty markets. Clustering was assessed visually by performing PCA on all of these six traits, and plotting scores along the first two PCs against each other to illustrate both the degree of clustering within classes, and the phenotypic “distance” between market classes.

### Experimental Design of the Half-Diallel

To demonstrate the utility of the phenotyping methodologies described here in the genetic analysis of root morphology, a half-diallel mating design was used to determine the heritability of digitally phenotyped traits. Seven inbred carrot lines and one open-pollinated variety were used as parents in this diallel. Two inbred lines (B2566 and L1408) were developed by the USDA-ARS Vegetable Crops Research Unit, which breeds primarily for the fresh market carrot industries (Simon et al., 1987); five inbreds (W279, W289, W287, W278, and W280) were produced by the University of Wisconsin-Madison carrot breeding program, which breeds primarily for processing (canning and juicing) industries (Goldman, 1996); the open-pollinated variety OSSI-Ball is a Parisienne-type grown for specialty markets (Luby and Goldman, 2016). Together, these eight accessions represent the most prevalent market classes in the United States, and consequently much of the diversity in root shapes that appear in public sector carrot breeding in the United States (Supplementary Figure 1). An additional benefit of using these inbred lines is that both sterile and fertile versions, relying on a cytoplasmic-genic male sterility system (Eisa and Wallace, 1969; Peterson and Simon, 1986), were available, facilitating the efficient production of hybrids. In the case of OSSI-Ball, only fertile roots were available, and thus this genotype was used as a male parent in each cross. A representative cross between an inbred line used in the breeding of fresh market types (L1408) and a specialty market type (OSSI-Ball) illustrates the typical manner in which root dimensions and shape phenotypes combined in crosses between divergent market class types (Supplementary Figure 2).

L1408 and B2566 were grown at Miller Farms in Hancock, WI (44°08′N, 89°32′W), and all other genotypes at Jack’s Pride Farm in Randolph, WI (43°37′N, 89°00′W) in the summers of 2017 and 2018. Harvested roots were vernalized at 4°C for 12 weeks with shoot growth removed before being planted in pots (15.2 cm tall × 13.8 cm diameter, filled with a blend of one-third Pro-Mix High Porosity (Premier Tech, Quakertown, PA) and two-thirds field soil). These vernalized roots were grown at 20°C with a 16 h photoperiod at the Walnut Street Greenhouses in Madison, WI. Following flowering, pairs of roots—one sterile, one fertile—were selected for crossing: immediately following the appearance of anthers, the umbels of each pair were enclosed in a cloth bag, to which blue bottle fly pupae were added at weekly intervals to ensure high rates of pollination. This process was carried out during both the winter of 2017–2018 and 2018–2019 in order to obtain progeny from each of the 28 pairwise crosses.

Following seed-set, umbels were separated, and seed was harvested by hand from the sterile parent. F_1_ seed from each cross was grown in two replicated 3 m plots at Jack’s Pride Farm in the summer of 2019. Roots were thinned to a density of 1 seedling per 5 cm 21 days after planting (DAP), and roots from each plot were harvested from the middle of each row 107 DAP. Fifteen to twenty roots were sampled from each replicate of each F_1_ progeny family in order to minimize bias as much as possible in the estimation of variances.

### Estimation of Heritabilities

For each of the two replicates of every F_1_ family, 15–20 roots were randomly selected for digital phenotyping. Because neither reciprocal crosses nor parental lines were included along with the F_1_ progeny, the Method IV, Model I diallel analysis was utilized, as described by Griffing (1956):

$$y_{i\,j\,k}=\mu +g_{i}+g_{j}+s_{i\,j}+\varepsilon_{i\,j\,k}$$

where μ is the population mean, *g_i* is general combining ability (GCA) effect of the *i*^*th*^ parent, *g_j* is the GCA of the *j*^*th*^ parent, *s*_*ij*_ is the specific combining ability (SCA) effect of the *ij*^*th*^ cross (where *s*_*ij*_=*s*_*ji*_), and ε_*ijk*_ is the residual error of the *ijk*^*th*^ root. All terms, excluding the residual error, were modeled as fixed effects.

Variance components for GCA, SCA, and error terms were calculated on the basis of expected mean squares:

$$E\,M\,S_{G\,C\,A}=V_{e}+V_{S\,C\,A}+\left(p-2\right)\,V_{G\,C\,A}$$

$$E\,M\,S_{S\,C\,A}=V_{e}+V_{S\,C\,A}$$

$$E\,M\,S\,\left(\varepsilon \right)=V_{e}$$

where *p* is the number of parental lines (in this study, 8), and *V_e* is equal to the error variance calculated on an entry-mean basis ($\frac{\sigma_{\varepsilon }^{2}}{r}$), with *r*, the number of replicates, equal to 2. Additive (*V_A*) and dominance (*V_D*) components were subsequently estimated as described by Pederson (1971):

$$V_{A}=\frac{4}{1+F}\,V_{G\,C\,A}$$

$$V_{D}=\frac{4}{{\left(1+F\right)}^{2}}\,V_{S\,C\,A}$$

Broad- (*H*^2^) and narrow-sense (*h*^2^) heritabilities were finally calculated as:

$$H^{2}=\frac{V_{G}}{V_{G}+\frac{V_{\varepsilon }}{r}}$$

$$h^{2}=\frac{V_{A}}{V_{G}+\frac{V_{\varepsilon }}{r}}$$

Where *r* = 2 replications; following Falconer (1996), total genotypic variance, *V_G*, was defined as *V*_*A*_+*V*_*D*_. Baker’s ratio was calculated as:

$$\frac{2\,M\,S_{G\,C\,A}}{2\,M\,S_{G\,C\,A}+M\,S_{S\,C\,A}}$$

As such, this metric ranges from 0 (in the case of all variance being attributed to SCA) to 1 (in the case of all variance being attributed to GCA) (Baker, 1978).

### Software Availability

Python code for the image acquisition platform and scripts for producing binary masks are available at: https://github.com/shbrainard/carrot-phenotyping. MATLAB algorithms for straightening binary masks and performing PCA on contours or curvature values are available at: https://github.com/jbustamante35/carrotsweeper.

## Results

### Accuracy of Image-Derived Phenotypes

**FIGURE 2:**
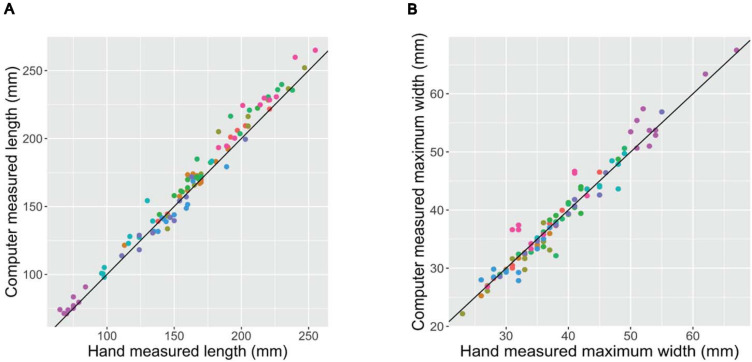
Hand- (*x*-axis) vs. digital image-based (*y*-axis) measurements of length **(A)** and maximum width **(B)** of 100 carrot roots. Each point represents a unique carrot; colors indicate 1 of 10 carrot accessions representing a variety of market classes. RMSE of the linear model *y* = *x* was 8.30 mm for length and 2.00 mm for maximum width.

Prior to a rigorous evaluation of any experimental populations, it is critical to confirm that a newly developed phenotyping platform produces accurate and reliable phenotypes. Scatter plots of the root phenotypes obtained from digital images vs. hand measurements confirms that the two methods provide highly consistent results (Figure 2). For both length and maximum width, both methods generated extremely similar measurements across the range of phenotypes measured. Root mean squared error (RMSE; the square root of the squared residuals) of the linear model *y* = *x* was 8.30 mm for length and 2.00 mm for maximum width.

In addition to this parity between human- and computer-based phenotypes, the variance associated with the arbitrary rotational aspect of a given carrot root beneath the camera was also evaluated. Some variance due to orientation should be expected, particularly in the estimation of maximum width, because this method analyzes a two-dimensional projection of a three-dimensional root, and carrots are not perfectly symmetric around their longitudinal axis. Pairwise comparisons were made between phenotypes extracted from three photos of individual, diverse roots that were variably rotated along this long axis. These comparisons demonstrated that deviation from symmetry about the long axis of the carrot was responsible for minimal variance in maximum width measurements and affected length even less so (Supplementary Figure 3). The variation between pairs of images was very similar to the variation between human and digital image-measurements presented in Figure 2. These results indicate that a single two-dimensional projection of a three-dimensional root is sufficient to obtain reliable estimates of key morphological phenotypes.

The diverse USDA-NPGS carrot collection was also evaluated in order to compare variation due to digital phenotyping error with variation between genotypes, which is typically the most relevant criteria from both a genetic and plant breeding perspective. Least significant differences (LSDs) between accessions were calculated for both length and maximum width, with *MS*_*Error*_ estimated by ANOVA (utilizing a linear model in which accession was included as a fixed effect). LSDs of 35.8 and 7.5 mm were calculated for length and maximum width, respectively (with α = 0.05). The measurement error associated with this digital phenotyping platform is thus many times smaller than the statistical threshold for distinguishing distinct carrot genotypes from each other.

### Principal Component Analysis of Contours

**FIGURE 3:**
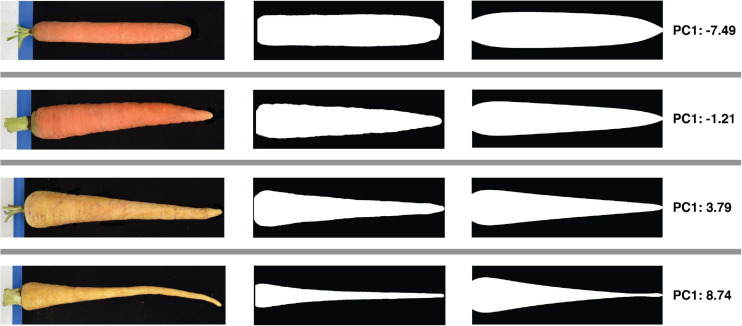
Quantification of size-independent variation in carrot root shape using PCA of length- and width- normalized contours. All carrots were standardized to a maximum width of 1 and a length of 1,000, and contours were decomposed into five principal components. Rows correspond to four representative roots sampled from each quartile of the range of scores along the first principal component, and illustrate the nature of the phenotypic variance captured by this first component. From left to right: raw color photos of roots taken during image acquisition; straightened binary masks of the corresponding root; simulated root profiles generated by taking the product of the first PC score pertaining to this root (far right) and the mean of all other PC scores with the transpose of the eigenvectors generated during eigendecomposition. These simulated profiles demonstrate that variation along the first principal component reflects the degree of root fill, or extent to which a carrot preserves its maximum width down its length.

An automated tool for simply measuring root length and maximum width would advance research into the genetic control of root development, but understanding the genetic variation that underlies variation between market classes requires additional information regarding shape in particular. A method was therefore developed to produce, for each root image, a set of contour points derived from width measurements made at 1,000 evenly spaced points along the long axis. Normalizing each contour dataset with respect to the maximum in both the *x* and *y* (width and length) dimensions, as described above, makes the shape information they contain comparable across the diverse sizes of cultivated accessions drawn from the USDA-NPGS carrot collection. A total of 8,687 images representing 683 accessions were collected and analyzed in this manner to produce a contour dataset for each root. Applying PCA to this set of contours showed that the first principal component (PC1) explained 84.9% of the total variance in the contours, with PC2 and PC3 explaining only 9.6 and 2.5% of the variance, respectively. The eigenvectors of this PCA were used to generate simulated root profiles based on specified PC scores. Figure 3 shows that decreasing the score along PC1 while holding all other PC scores equal to their mean reduces the rate of taper toward the tip, i.e., the roots with lower PC1 scores maintain their maximum width further along their lengths compared to roots with a higher PC1 score. To the best of our knowledge, this study represents the first quantitative assessment of this aspect of carrot root morphology; despite its importance in accounting for the vast majority of size-independent variation in carrot root shape, this trait—which is referred to as “root fill” in subsequent analyses—has not been previously described. This is likely a function of its convolution with carrot size, and as such, this analysis demonstrates the power of digital image phenotyping in allowing for the quantification of traits that cannot be evaluated by hand or by eye.

### Principal Component Analysis of Shoulder and Tip Curvatures

**FIGURE 4:**
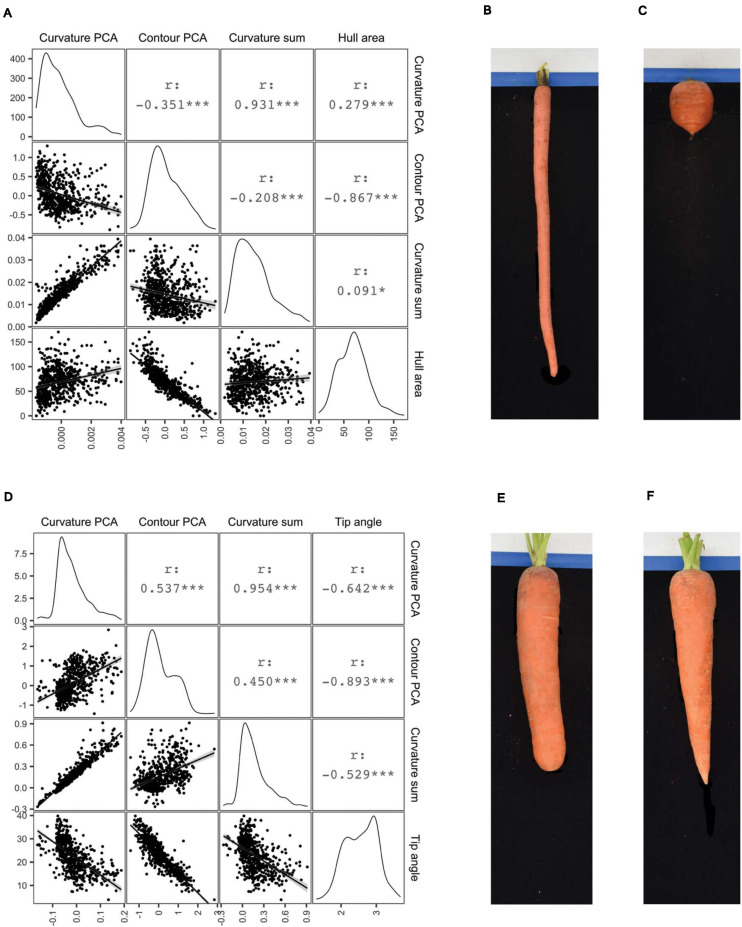
Correlograms comparing several measures of shoulder shape **(A)** and tip shape **(D)**: PC1 scores derived from curvature values (“Curvature PCA”), PC1 scores derive from normalized contours (“Contour PCA”), the sum of curvature values, and either shoulder hull area **(A)** or tip angle **(D)**. For Pearson correlation coefficients (*r*) shown above the diagonal, ***p < 0.001; *p < 0.1. Images on right illustrate representative roots drawn from the extremes of the first principal component scores corresponding to shoulder curvature values **(B,C)** and tip curvature values **(E,F)**.

The PCA of whole-root contours presented above did not require pre-determining what aspect of shape contributes most significantly to variation across this carrot population. This can be contrasted with deliberately measuring shapes of interest, such as the distribution of curvature in the shoulder and tip regions that humans subjectively consider when distinguishing cultivars from differing market classes. Algorithms to perform such phenotyping were included in the platform described here, and as such, this study represents the first to rigorously evaluate variation in curvature in the shoulder and tips of carrot roots in a quantitative manner. The curvature quantified in this case is the instantaneous rate of change of angle of the vector normal to the contour as this vector moves along the contour. Put another way, curvature at each contour point is proportional to the reciprocal of the radius of the circle that is tangent to the contour at that point. After fitting smoothed splines to the top 50 (for measuring the root shoulder) and bottom 50 (for measuring the root tip) contour points, curvature at each point was calculated to construct the respective covariance matrices needed to apply PCA. PC1 of the shoulder curvature values explained 87.3% of the total variation in this region, while PC1 of the tip curvature values explained 84.2% of their total variation. Representative examples of roots with PC1 values in the 1st and 99th percentiles of shoulder curvatures are shown in Figures 4B,C. The Imperator type (Figure 4B) has almost no curvature in the shoulder, while the broadly shouldered Parisienne type (Figure 4C) exhibits much more substantial curvature. Representative examples of roots with PC1 values in the 1st and 99th percentiles of tip curvatures are shown in Figures 4E,F. The extremely blunt-tipped Nantes type in Figure 4E can be contrasted with the highly acuminate Danvers type in Figure 4F.

Figures 4A,D show correlograms of these curvature-derived metrics, with alternative methods of measuring shoulder and tip shape: PC scores derived from curvature values (“Curvature PCA”), PC scores derive from normalized contours (“Contour PCA”), the sum of curvature values, and either shoulder hull area or tip angle. As would be expected, PC scores derived from curvature values are strongly correlated with the sum of curvatures in the shoulders (*r* = 0.931) and tips (*r* = 0.954). Less expected was the poor correlation between curvature PC scores and contour PC scores derived from the same region (*r* = –0.351 for the shoulder region and *r* = 0.537 for the tip region). This suggests that the process of fitting smoothed splines to the contours gives rise to a meaningful difference between the phenotypes that are measured by quantifying “variation in the curvature” and “variation in the contour” of the shoulder and tip regions.

In general, it is also clear that for any particular pair of phenotypes, the correlations are larger in the tip region, compared to the shoulders. In particular, while tip angle appears to be a moderately accurate surrogate measure for variation in tip curvature, hull area has a relatively weak correlation with shoulder curvature. This is logical, since broadly shouldered carrots and carrots completely lacking shoulder curvature both exhibit large hull areas, whereas tip angle varies monotonically with the curvature of the tip.

### Cluster Analysis of Representative Carrot Market Class Types

**FIGURE 5:**
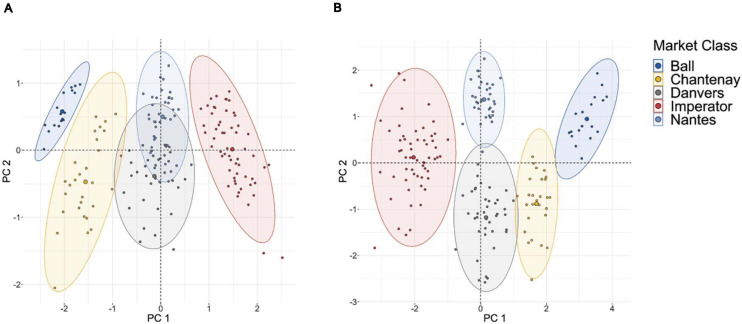
**(A)** PCA-based clustering of 175 roots sampled from five major market classes on the basis of only length and maximum width; **(B)** PCA-based clustering of these same roots using PCA of curvature values in the tip and shoulder, root length, maximum width, aspect ratio, and root fill.

The results of the clustering analysis using five diverse market classes are shown in Figure 5B. Clear clustering is observed for all five market classes, indicating that this phenotyping pipeline effectively characterizes the key phenotypic components of market classes. This can be compared to a clustering analysis which uses only those two traits most readily measured by hand: length and maximum width (Figure 5A). While the most phenotypically divergent market classes are still distinguishable (e.g., Imperator and Parisienne), the exclusion of shape descriptors markedly increases the overlap between market classes that are similar in their overall dimensions (e.g., Nantes and Danvers, which differ primarily only in tip fill). This confirms that the phenotyping platform measures shape parameters that parallel the morphological differences between market classes.

### Diallel Mating Design

All pairwise crosses were made between eight parental lines to construct a half-diallel population. Seven hundred sixty F_1_ roots drawn from this set of progenies were phenotyped using the pipeline described above. Mean length was 18.12 cm, with a standard deviation of 4.46 cm; mean maximum width was 4.72 cm with a standard deviation of 0.80 cm; and mean L/W ratio was 4.01 with a standard deviation of 1.34. For all traits, MS_*GCA*_ was larger than MS_*SCA*_, although the degree to which this was the case varied substantially from phenotype to phenotype, from over 15× greater in the case of L/W ratio, to only 1.43× greater in the case of total root size (Table 1). This finding is captured well in Baker’s ratio: in general, values were found to be close to unity, suggesting meaningful degrees of additive gene action for all traits considered.

**TABLE 1:** Parameters estimated from ANOVA of the half-diallel using Griffing’s Method IV, Model 1. Variance components and heritabilities are reported for the primary size and shape traits which define market class.

	**Length**	**Width**	**L/W**	**Root size**	**Root fill**	**Tip curvature**	**Shoulder curvature**
	**(mm)**	**(mm)**	**Ratio**	**(mm^2^)**	**(PC score)**	**(PC score)**	**(PC score)**
MS_*GCA*_^†^	5200.03	175.14	5.84	2.79	1.77	1.18e-04	5.16e-05
V_*A*_	1445.25	50.36	1.82	0.28	0.37	1.57e-05	1.54e-05
MS_*SCA*_^†^	864.29	24.05	0.38	1.94	0.67	7.28e-05	5.41e-06
V_*D*_	651.05	15.94	0.33	1.43	0.55	1.83e-05	4.30e-06
V_ε_	213.24	8.11	0.06	0.51	0.12	5.45e-05	1.07e-06
*H*^2^	0.95	0.94	0.98	0.87	0.94	0.55	0.97
*h*^2^	0.66	0.72	0.84	0.14	0.38	0.26	0.76
*BR*^‡^	0.92	0.94	0.97	0.74	0.84	0.77	0.95

*^†^Mean sum of squares from the ANOVA. ^‡^Baker’s Ratio.*

Broad-sense heritability values were ≥0.94 for all traits except total root size and tip curvature. This somewhat surprising finding indicates a high degree of genetic influence over phenotypes that are exposed to a great degree of environmental variability, due to roots’ direct contact with the inherently heterogeneous soil profile. In this regard, two factors should be kept in mind: first, the soils in which this trial was grown is a Houghton muck, a deep saprist histosol with more than 50% organic matter. Given the aggressive tillage prior to seed bed preparation, this leads to a highly uniform soil profile with minimal compaction. Together with conventional weed control and fertilizer application, this produces one of the most uniform environments for growing root crops in Wisconsin. Thus, it is somewhat unsurprising that environmental variation was minimized, and heritability maximized, in such a production system. Secondly, the fact that seven of the eight parents in this diallel were inbred lines likely contributed to phenotypic uniformity within full-sib families due to the genetic uniformity within each particular hybrid combination.

Narrow sense heritabilities displayed a wide range of values, from 0.14 for total root size, to 0.84 for L/W ratio. Length, maximum width, and shoulder curvature all exhibited intermediate values (0.66, 0.72, and 0.76, respectively). In general, these values conform to the success of modern breeding, which has led to the development of highly typified long and slender carrots for the fresh market, and much shorter, broader, heavily tapered carrots for the processing industries.

It is interesting to consider why root size and aspect ratio—both traits that would intuitively be understood as primarily functions of the overall dimensions of the root (length and maximum width)—possess such markedly different narrow-sense heritabilities. While aspect ratio is primarily the function of two highly heritable traits (length and width), root size is additionally a function of root fill, and in this particular population, the correlation between maximum width and root fill is weak (0.12). That is, whether a carrot has a wide shoulder or narrow shoulder is not highly correlated with widths elsewhere along the carrot.

## Discussion

### The Quantitative Traits Underlying Market Class

It is relatively straightforward to enumerate the suite of parameters that are involved in defining a given root’s market class. However, it has historically been challenging to quantify these parameters in a manner independent of each other, integrate them into a single metric to determine which classes are most similar to each other, or evaluate how much variation exists within a given class. The novel phenotyping platform presented in this study overcomes these challenges.

The starting point for this workflow was the methodology developed by Turner et al. (2018), which demonstrated that binary masks of whole-carrot images could be used to extract phenotypes corresponding to morphological attributes of both the root and shoot. However, because that study was not specifically focused on root morphology, several improvements were made in the development of this novel pipeline. In Turner et al. (2018), imprecision in the separation of root and shoot tissue prevented phenotyping of the root shoulder, and a lack of pre-processing algorithms to straighten and de-tip carrot roots limited the phenotyping of the carrot tip. Refinements of the image acquisition process, and implementation of standardization steps prior to phenotyping in the platform presented here addressed both of these issues. As shown in the clustering analysis above, both the shoulder and tip phenotypes this pipeline is able to measure are critical in quantifying the components of market class. In addition, by controlling for these additional sources of variation, the pipeline reported here was able to utilize PCA of the entire root profile to identify a novel trait: the first principal component of the straightened, length- and width-normalized contours, or what has been referred to above as “root fill.” This previously undescribed trait accounts for over 80% of the size-independent variation in carrot roots, and this phenotyping pipeline now provides breeders and researchers with a method for visualizing and quantifying a source of shape variation that would otherwise be confounded with roots’ dimensions.

Physiologically, carrot roots—like many plant storage roots—are formed through a swelling of the taproot prior to dormancy, which is driven by the production of supernumerary cambia, i.e., secondary growth characterized by the formation and expansion of additional xylem and phloem tissues (Goldman, 2020). As such, the phenotyping methods described here—and the quantification of root fill in particular—hold significant potential not only in the context of plant breeding, but in understanding plant tissues that have been modified through domestication. Precisely studying variation across carrot market classes therefore represents a unique opportunity to deepen our understanding of the genetic bases of secondary root development in general. Future studies based on linkage mapping populations or association panels could utilize this phenotyping pipeline to identify QTL associated with these underlying physiological processes.

Furthermore, because this platform is not designed around a machine learning algorithm for classifying carrots into predefined—and therefore static—market classes, it can be adapted to any range of root shapes. This is particularly useful for a character like market class, which is determined by current agricultural practices and culinary preferences. While these classes are therefore malleable, and will certainly change over time, their component phenotypes will still be quantifiable by way of the pipeline described here. This is illustrated well by the PCA-based clustering analysis, which clearly identified clusters corresponding to five major market classes using only their component phenotypes.

### Trait Heritabilities in a Diallel Mating Design

To demonstrate this phenotyping platform’s utility in the context of a genetic analysis that is particularly relevant from a breeding perspective, a novel diallel experiment was conducted, drawing parental material from all of the predominant United States market classes. The results of this analysis are striking: both the size and shape phenotypes underlying market class were shown to be largely controlled by additive gene action. From a practical perspective, the relatively high narrow-sense heritabilities reported here is a reflection of breeders’ success in efficiently selecting for these traits. From a genetic perspective, however, this finding does not necessarily indicate simple control, in the sense that these traits are necessarily primarily controlled by only a few, large-effect quantitative trait loci (QTL). Large additive genetic variance components could also be associated with highly polygenic traits that simply lack dominance and epistatic variance (Huang and Mackay, 2016).

It is also important to note that the heritabilities reported here reflect phenotypes measured in a single—albeit common and economically important—environment in Wisconsin. Thus while they are quite high, and wider, multi-environment evaluation of heritabilities is therefore warranted, the values reported here likely represent a ceiling for heritabilities calculated across multiple environments. However, the precise degree to which multi-environment trials might lead to lower estimates of heritability is not clear, given the fact that the estimates reported here accord with values previously estimated for root length (Prasad and Prasad, 1980; Brar and Sukhija, 1981). Additionally, it is important to stress that heritability is a parameter of the population under consideration, and not purely a function of the trait it describes. Estimates of heritability will vary depending on the particular cultivars or population studied, as has been found in carrot with respect to heritabilities for nematode resistance (Huang et al., 1986; Vieira et al., 2003) as well carotenoid concentrations (Fernandes Santos and Simon, 2006). Because the parents used in this diallel represent a diverse set of inbred lines drawn from United States breeding programs, these results will immediately aide breeders in selecting specific hybrid combinations, depending on the precise market class being targeted. At the same time, however, it is important to recognize that these inbred lines were intentionally drawn from contrasting market classes, and thus produced progeny families with extreme levels of phenotypic variance. The diversity observed in these inter-market class crosses may therefore exceed what breeders observe in practical contexts, involving a crosses within a more narrow subset of germplasm.

### Potential of Digital Image-Based Phenotyping

Beyond its utility in simply describing phenotypic variability, the automated potential of the phenotyping platform presented here has substantial promise within plant breeding programs, where the resources required to screen large populations for a given set of traits is often a key factor limiting population sizes. From this perspective, the pre-processing and image analysis stages of this pipeline are already explicitly automated. Furthermore, the acquisition algorithms described here are robust to many horticultural and agronomic plant structures; only the specific RGB thresholding indexes used to distinguish plant tissue from background require adjustment to allow for the accurate production of a binary mask. With regards to the pre-processing and analysis algorithms, the only requirement to their broader application is that the phenotyped object be non-branching. While this excludes, for example, wild carrot (which is typically highly branched), within horticultural and agronomic crops this would allow for the phenotyping of a wide array of fruit, roots and tubers.

A critical determinant of the potential practical utility of the digital phenotyping platform described here is the rate at which phenotypes can be obtained; this rate is in turn a function of two components: the rate at which images of carrot roots are acquired, and the computational time required to extract and record phenotypes from these images. In this study, images containing two carrots and two QR codes required one person working for 2 min to acquire, or 1 min per carrot. An additional 1 min of computational time was required to perform pre-processing of the binary masks produced during acquisition, and phenotyping of these standardized images using a MacBook Pro with a 3.3 GHz Intel Dual-Core i7 CPU and 16 GB of 2,133 MHz LPDDR3 RAM. Importantly, these MATLAB and Python scripts can process images in bulk without any human intervention, and thus this computational time does not involve any additional labor time.

As a result, we consider this platform to be immediately usable for many research applications. From a more applied perspective, it is unlikely that all stages of population and or inbred line development within commercial breeding programs would benefit from the precision provided by this platform, to a degree that outweighs the additional labor required. Specific use cases, however, such as the development of genomic prediction models, which require very precise, quantitative phenotypic measurements for only a defined subset of germplasm, could productively utilize these methods.

We hope that the digital phenotyping workflow described here lead to further improvements in both the acquisition and phenotyping stages of digital image analysis, enabling further expansion of the utility of such approaches in both scientific and applied domains.

## Data Availability Statement

The datasets presented in this study can be found online at the Harvard Dataverse Repository: doi.org/10.7910/DVN/LOVXZA.

## Author Contributions

SHB, JCD, and ILG designed the experiments. SHB performed the experiments, analyzed the data, and prepared the manuscript. SHB, JAB, and EPS designed the phenotyping platform. All authors have read and approved the manuscript.

## Conflict of Interest

The authors declare that the research was conducted in the absence of any commercial or financial relationships that could be construed as a potential conflict of interest.
