# Supplementary material for: A Digital Image-Based Phenotyping Platform for Analyzing Root Shape Attributes in Carrot
Source: Front Plant Sci. 2021 Jun 16;12:690031. doi: 10.3389/fpls.2021.690031 (PMC8244657; doi:10.3389/fpls.2021.690031)
Supplement: Supplementary file 1 [file Data_Sheet_1.docx]

Supplementary Material

# Supplementary Figures


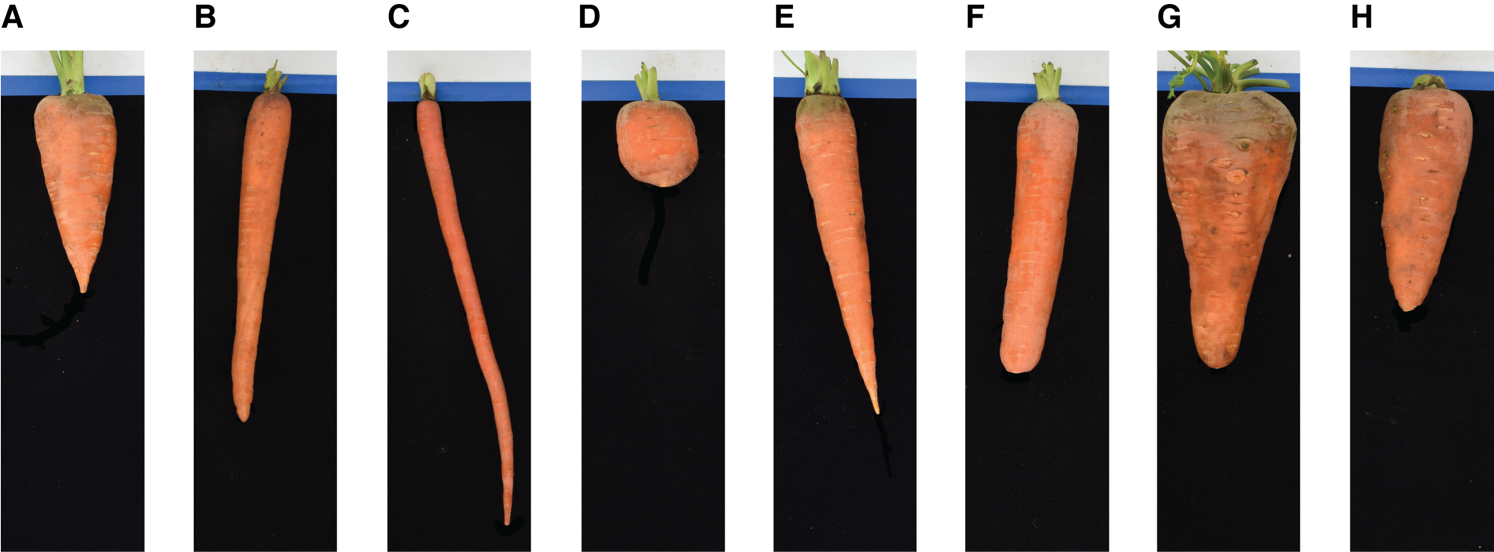


**Supplementary Figure 1.** Carrot genotypes used in the half-diallel mating scheme. **(A)** W279; **(B)** B2566; **(C)** L1408; **(D)** OSSI-Ball; **(E)** W289; **(F)** W287; **(G)** W278; **(H)** W280. A, G & H exemplify the Chantenay-type processing carrot; E & F typify the Danvers, and Nantes market classes, and typically sold as fresh-market or storage carrots; D is a specialty Parisienne (or Ball) type; and C is an Imperator type used in the production of baby carrots.

**
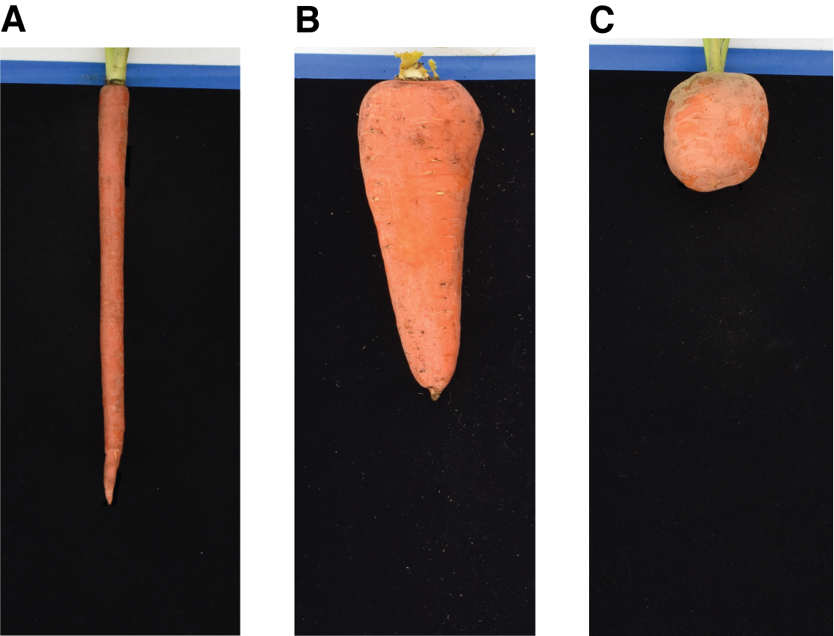
**

**Supplementary Figure 2.** Representative roots from one of the pair-wise crosses included in the diallel mating scheme: **(A)** L1408; **(B)** L1408xOSSI-Ball; **(C)** OSSI-Ball.

**
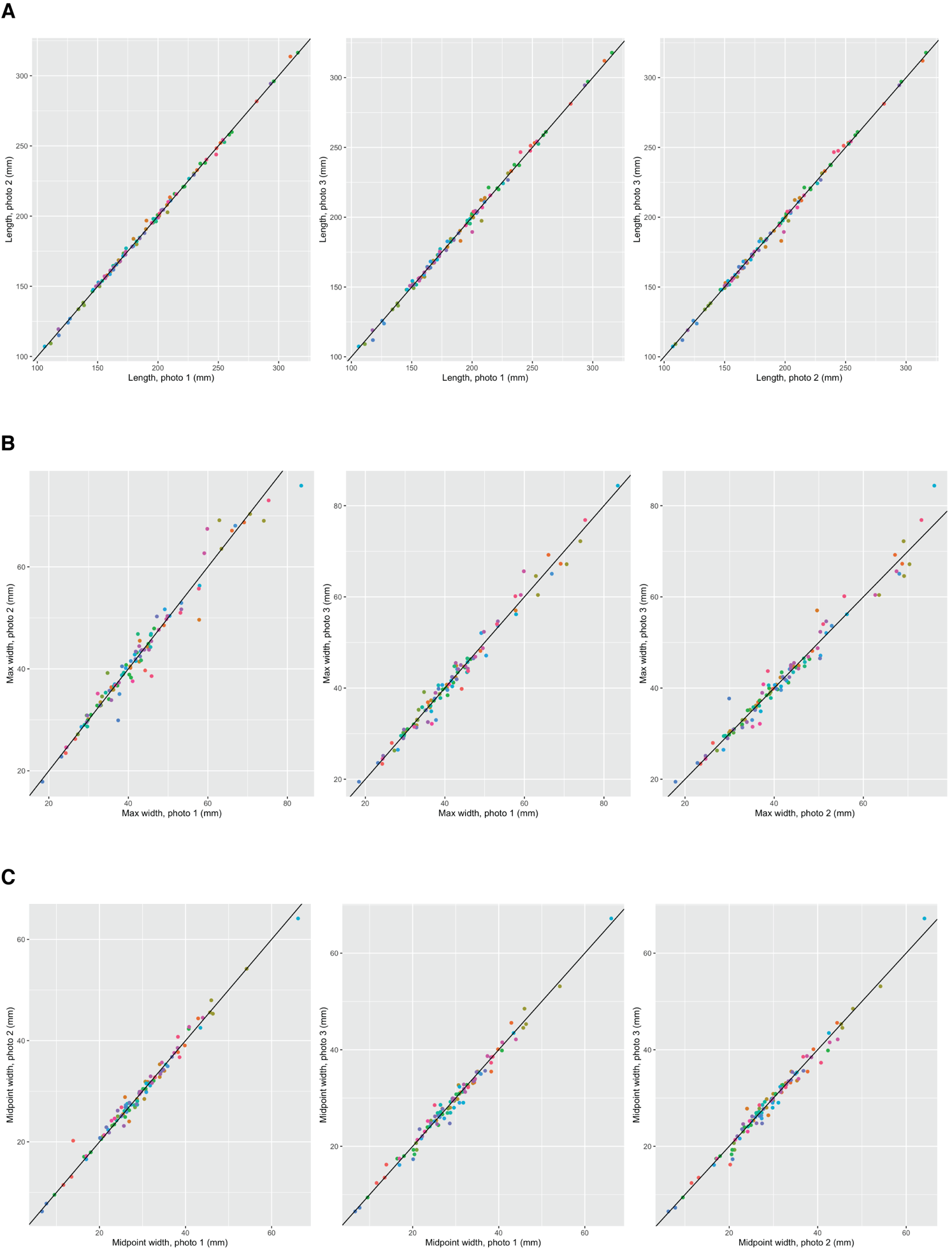
**

**Supplementary Figure 3.﻿** Pairwise comparisons of phenotypes **(A)** length; **(B)** maximum width; **(C)** midpoint width, extracted from multiple photos of individual roots. Each point represents a single carrot that was photographed three times from three different angles (as in Fig. 3, color corresponds to distinct genotypes). Left-most panels compare photo 2 against photo 1, middle panels photo 3 versus photo 1, and right-most panels photo 3 versus photo 2.
